# ‘SHE'S MY SISTER-IN-LAW, MY VISITOR, MY FRIEND’ – CHALLENGES OF STAFF IDENTITY IN HOME FOLLOW-UP IN AN HIV TRIAL IN WESTERN KENYA

**DOI:** 10.1111/dewb.12019

**Published:** 2013-03-22

**Authors:** Philister Adhiambo Madiega, Gemma Jones, Ruth Jane Prince, Paul Wenzel Geissler

**Keywords:** HIV, Kenya, Africa, clinical trials, global health research, community relations, research ethics, research subjects, fieldworkers

## Abstract

Identities ascribed to research staff in face-to-face encounters with participants have been raised as key ethical challenge in transnational health research. ‘Misattributed’ identities that do not just deviate from researchers' self-image, but obscure unequivocal aspects of researcher identity – e.g. that they are researchers – are a case of such ethical problem. Yet, the reasonable expectation of unconcealed identity can conflict with another ethical premise: confidentiality; this poses challenges to staff visiting participants at home. We explore these around a case study of ‘follow-up’ staff, observed during an ethnographic study of a Kenyan HIV ‘trial community’, which included participant observation, conversations, and interviews with staff (n = 79) and participants (n = 89). We found that because of the need to maintain confidentiality and because of some suspicions towards researchers, research staff drew upon alternative identities – presenting themselves to non-participants as relatives or friends, rather than as researchers. Several staff experienced this as necessary but uncomfortable. Simultaneously, staff and participants forged close relations in line with their fictional identities, which however also posed challenges because they entailed personal responsibilities that were difficult to live up to, due to limited resources, and the trial's limited duration. Similar challenges may arise in transnational HIV treatment programmes and should be explored further in that context.

## Introduction

Population-based HIV science is an important part of medical research in sub-Saharan Africa.^1^ Apart from research – epitomised by clinical trials – large-scale HIV interventions, funded by international donors and often involving transnational academic institutions, are innovative undertakings that also rely upon systematic practices of surveillance, data collection and evaluation. Together, HIV research and interventions shape a creative domain of medical science in Africa, which generates new knowledge and new social practices, and which involves increasing numbers of people as patients or study subjects, as well as staff and volunteers. In doing so, HIV science has to deal with stigma and the rights-based emphasis on confidentiality,^2^ and often provokes concerns and critical responses in the communities involved.^3^

In this context, the issue of ascribed ‘researcher identity’ that Simon and Mosavel recently brought to attention is of particular importance, providing new perspectives on familiar ethics concerns about information, choice and justice.^4^ Identity, including ideas about origins, motives and capacity, knowledge (and ignorance) of the other, and meanings attributed to the relation with the other, bring to the fore the importance, for the research encounter, of fieldworkers or first-line research staff, who engage regularly, face-to-face and over a period of time, with participants and patients. Research fieldworkers confront issues arising from ascriptions of identity especially when they encounter research participants, relatives and other community members outside the health facility or research clinic.

Recent social studies of African clinical research have drawn attention to the obvious importance of fieldworkers in the production of scientific data, and of personal relations between staff and participants for the success of medical research and interventions, drawing attention to dimensions like trust and friendship.^5^ This paper contributes to this literature. It focuses on ‘home follow-up’ in an HIV research study, i.e. procedures by which participants, which previously had consented to and commenced participation in an HIV clinical trial, were traced in their urban and/or rural communities. The primary purpose of these visits was to assess health, observe for adverse events due to study drugs, deliver medicines, support adherence to trial regimes, call back for repeat or management of abnormal laboratory results, monitor growth in children through anthropometrical measures, and trace participants who had missed study appointments in the research clinic.

Instead of patients coming to a health facility to receive care, ‘home follow-up’ could be said to invert the usual direction of care: staff, who were trained by the trial to conduct specific health related activities, proactively follow patients – often asymptomatic – up to their homes, bringing medical attention and trial-related health care into the private sphere. The experiences of such follow-up activities in HIV research are of interest beyond the purview of clinical trials work, because follow-up by fieldworker-type non-medical staff (referred to, e.g., as ‘volunteers’, ‘peer educators’ ‘TB ambassadors’, ‘cough monitors’ or ‘defaulter tracers’) are also a pillar of contemporary HIV care and treatment (and related, e.g. TB) interventions, which require strict patient adherence to treatment over extended periods of time. Follow-up serves here to evaluate and reinforce adherence in order to achieve success in treatment and minimize development of antibiotic or antiretroviral resistance, and to help maintain participants or patients in research/treatment programs. Maintaining participation in research trials and in health interventions is an important aspect for determining the impact of new interventions and in assuring continued funding for intervention programs. The experiences from follow-up in HIV research, described below, may therefore also be of interest for HIV/AIDS programmes.

We focus here on an intervention trial, conducted between 2005 and 2009, which assessed the potential of an approved anti-retroviral therapy regime to prevent mother-to-child-transmission of HIV through during pregnancy and breastfeeding. The study was successful: the intervention reduced HIV transmission to infants and had scientific and policy impact.^6^ Among our interviewees, the trial was widely praised for the positive social relations it had engendered between research staff and research participants, based on good services, clinical commitment and personal trust. This trust was to no little extent of the achievement of follow-up staff. However, there have been few studies of the actual practices of ‘follow-up’ staff and the challenges facing them regarding trust, responsibility, and confidentiality, and how to balance these concerns with following study protocol and achieving scientific data. This paper addresses this gap.

## Methods

Data collection was part of a three-year ethnographic study of the ‘trial community’ of HIV research – scientists, clinicians, managers, laboratory and field staff, volunteers, trial participants, community members etc – in a research site run by the Kenyan Medical Research Institute (KEMRI) in collaboration with the US Centres for Disease Control and Prevention (CDC). It included participant observation during work in- and outside the research clinic, informal conversations, and audio-recorded interviews with staff (n = 79) – of which 9 were designated follow-up staff – and trial participants (n = 89). Among staff, all staff with connection to the above mentioned PMTCT trial were interviewed with the exception of a small group who opted out of the study. Interviews often stretched over several sessions of up to two hours each, and evolved, following only a very rough guide, from biographical information, current living circumstances and plans for the future, through detailed accounts of and reflections on work practices, to perception of research, collaboration and its wider social and political context. All follow-up staff were interviewed repeatedly; they were also extensively followed by the authors during their daily work. This shed – in view of some authors' complexion – additional light on the challenges of confidentiality and identity and was particularly valuable for this paper.

Participants of the PMTCT trial were sampled for interviewing, observation and visits by snowballing through study staff and other participants; none of the participants approached refused to join our ethnographic study; some were followed for up to four years. Recruitment of participants commenced in the second half of the trial and was therefore biased towards those who joined the trial later; this might have affected the practices and skills of staff and their familiarity with participants, leading to more clearly established work practices; it might also have contributed to some degree of evolution from initial standard operational procedures. However, this does not affect the validity of findings and conclusions detailed below. In this paper we draw on a small part of these data, concerned with the practice of ‘home follow up’; we focus on one composite case study, representative of the ethnographic material, which is consolidated by observations and interviews with staff and participants.

The purpose of the ethnographic study was explained to all potential participants and all were given the opportunity to either verbally consent or opt out of observation. All participants in formal interviews and group discussions gave informed, written, consent. The study was approved by the ethical review committees of the involved institutions (IRB numbers: KEMRI 1080, CDC 5093, LSHTM 4057).

## Findings and Discussion

### Follow-up in an HIV trial

In the PMTCT trial we studied, approximately 500 participants were recruited upon HIV diagnosis at the local public hospitals' antenatal care units, and subsequent screening for social and medical criteria). Participants were required to adhere to a triple anti-retroviral regimen from late pregnancy (34–36 weeks gestation) to 6 months postpartum while they exclusively breastfed. At 6 months ARVs and breastfeeding were discontinued. If the woman met WHO criteria for treatment then the ARVs were continued. The participants were followed for 24 months during which they were expected to attend the research clinic adjacent to the Nyanza Provincial General Hospital, Kisumu, at varying intervals. The final outcome was the infant's HIV status.

Like all population based trials, the study's validity depended upon adherence to the study intervention and the study schedule of visits. Participants knew that: ‘it is necessary you follow the study rules’; staff appreciated participants who did so, and described them as ‘good participant’ – a term that was commonly employed in contrast to ‘difficult’ participants. The nine full-time staff of the ‘follow-up’ department spent most of their time outside the clinic, tracing lost participants and visiting others, to ensure adherence to study drugs and interventions, and clinic attendance. Follow-up visits also served to collect socio-economic and hygiene data and vital signs for mother and babies, conduct pill-counts (and ART syrup measurements) reflecting drug intake, and monitor adverse events.

All follow-up staff were women, and apart from the supervisor, non-medical with secondary school or certificate or diploma level education. They relied heavily on their job experience in preceding research or community health projects.^7^ Their salary was less than that of technically or medically qualified staff members within the organisation, but more than that of peers in other employment, such as public workers without higher degrees, or the staff of non-governmental development and health programmes. In terms of their gender, age, educational background, language and residential area, follow-up staff resembled the study participants, with the crucial difference that few of the latter had regular employment. The follow-up group was thus able to form a bridge between the scientifically or medically qualified research staff, and the participants, which was important for the successful conduct of the trial. As one follow-up staff noted: ‘they [the research team] take follow-up staffs like, you know, their tools; they are the toolbox of the study.’ The following account of a day's work of a follow-up staff shows some of the procedures, as well as the challenges this work involved.

**A follow-up day**

*Agwata*^8^*, an experienced follow-up staff, leaves the research clinic at 8 a.m. to follow one of her participants, Ajwang', who relocated after her husband rejected her and their baby upon finding out she was HIV positive. Ajwang' has nowhere to go to and keeps moving, like many of her fellow research participants: she cannot afford to rent a place of her own; her father, following local custom, does not want a mature daughter to live in his rural home; and neither is she welcomed in her younger sister's marital home where she is seen as an economic burden. For the time being, she rents a one-room semi-permanent house in a remote shopping centre and tries to set up a tailoring business. Her baby daughter is also HIV positive and both are entitled to free health care as part of the trial into which the daughter was born. Ajwang' has not visited the research clinic for a while and her final study visit is overdue.*

Ajwang' has no telephone so Agwata has spent two days tracking her through the relatives listed as contacts in her ‘locator form’ – a document completed upon recruitment of each new participant and filed in the follow-up staff's dedicated office in Kisumu. Agwata travels to the field in a land-cruiser bearing the name of the research organisation. Reaching the shopping centre where she was told Ajwang' was staying, Agwata gets out of the car and asks the shopkeepers if they know MamaAbongo, as she would be known here, but in vain.

A healthworker from the adjacent dispensary joins in the conversation. He takes Agwata to a lady from the same ethnic group as Ajwang' and Agwata, who stays nearby. At first she, too, denies knowing Ajwang'. Eventually she explains that the girl used to live next door but was chased away because of rumours that she was HIV positive. When Agwata asks the lady to help her find Ajwang', she claims that the place is far, and it is muddy, but Agwata convinces her by tracing their shared clanship links and by joking that she won't take her blood, referring to a widespread rumour about medical research workers.

The home is ‘inside’, off the road, and Agwata resorts to walking, guided by a girl recruited from a homestead. The terrain is rocky and bushy and the sun stands high. Seeing Agwata stumbling along in her town shoes, the young girl gives her her flip-flops and continues on bare feet. They reach the home, but the house is locked. A neighbour tells them Ajwang' just left for her maternal home, half way back to where they came from that morning. Agwata walks back to the car, which meanwhile has found a way to follow her, and calls the research centre for further information.

After driving two more hours they arrive at the shopping centre near Ajwang's maternal home. Agwata meets an old lady who accepts a lift to the home. They find Ajwang' and her baby. “Sasa Mum” [All right, mum?] she greets Agwata, and they hug. There are many people in the house and since Agwata isn't sure whether Ajwang' has disclosed her HIV status, she hesitates to introduce herself (and the anthropologists). Ajwang' tells the household that she is her sister-in-law, and signals Agwata to come outside to chat. Agwata asks general questions about the baby's health, but she cannot examine or weigh her, nor count the mother's ARV pills or measure the baby's ARV syrup to check treatment adherence. The forms she brought remain incomplete. The research team has spent about 2,500 KSH (£20) on petrol alone (apart from salaries and allowances for her and the driver) – enough to feed mother and baby for a month. The baby seems a bit sick so Agwata gives the mother a referral sheet and money for the bus to bring the baby to the clinic the next day. She adds some small banknote ‘for the baby’. Ajwang' hides both in her bra.

For two days Agwata waits for Ajwang' to come to the clinic. She discusses her worries with the follow-up colleagues: ‘Our baby might be really sick, I'm afraid the mother is not making sure she adheres to the syrup’. She requests a car to go back to her home the following day, but the next morning Ajwang' arrives at the clinic. Rain had made the paths impassable, and she had not been able to phone. She completes her ‘exit’ visit from the trial and leaves the clinic with referral letters for herself and her daughter, for continued care and treatment at her local HIV patient support centre. Bidding farewell to Agwata, she waves: ‘see you’.

### Efforts

‘Going to the field’ requires resources: a vehicle and petrol, salary and allowances, bus fares, mobile phone credit and money in cases of emergencies. It also depends upon infrastructures like the regularly updated ‘locator form’ and the ‘encounters log’ in which follow-up staff record, for their internal communication, details of visits and challenges they met, such as curious neighbours or hostile husbands, which require particularly careful approaches. The locator form includes personalised maps of the participant's place and other locations where she may be found, detailed itineraries including bus stops, shops and other landmarks, the names of people to ask along the way, the participant's local nicknames, whether and to whom she had disclosed her HIV positive status, and phone numbers of her confidants. During Agwata's visit to Ajwang', telephone conversations with the records manager at the research clinic served to retrieve (and add to) the locator form information; other telephone calls to follow-up colleagues drew upon their informal knowledge of participants and local geography.

Despite motivated staff and excellent logistics, follow-up is challenging. It entails long working-hours and patience, physical effort to negotiate challenging terrain, threats from dogs, violent husbands and relations, demands for money and support in the face of abject poverty and hunger, and the risk of travelling in vain, because of participants' movements. Although the trial had only recruited mothers living in the city, many participants moved several times over the course of the trial. Driven by poverty, marriage instability and kinship rules, and under the strain of HIV suspicions or a positive diagnosis, this mobility is characteristic for young women in Western Kenya, many of whom ‘move around’, literally and in the local sense of the word: associating with changing male partners. These movements were the principal challenge of follow-up staff, because it entailed long journeys, often in vain, and forced them to relate and adjust to new social contexts.

### Professional identity

Another challenge of this work pertains to the identity of follow-up staff vis-á-vis participants and others, and the nature of their relationship. Belonging to a major transnational science organisation is not without ambiguities. Staff members were proud of this attachment, and the organisation was generally associated with expertise, access to medical treatments and technology, global connections, benefits and wealth. These associations, and people's trust and expectations were favourable when participants were to be mobilised, and when large cars, and visible car stickers and personal identity cards enhanced people's interest and expectations.

However, the global power and superior resources of the research institution, and some of its practices, notably blood specimen collection, also gave rise to concerns among the community and sometimes even to negative rumours about nefarious practices, like those described in social science literature on clinical research in Africa.^9^ Accordingly, also the follow-up staff in our HIV study had occasionally been accused – sometimes seriously, sometimes in a jest – as ‘devil worshippers’ or ‘blood suckers’, and although these confrontations were uncommon, all staff knew about them and they formed the backdrop for discussions and certain fieldwork practices.

These accusations did not arise directly from this study but they have a long history, and are part of a long-standing local idiom, which was widely known, but which only occasionally led to fears and anxieties.^10^ Importantly, these rumours and events were also familiar to staff and influenced their perception of risk in relation to fieldwork, and their conception of ‘the community’ from which participants were recruited (as a result staff were expected to conduct home visits in teams of at least two people). Even if concerns were not voiced in such exotic idiom, the disproportionate resources displayed and deployed during follow-up, in pursuit of treating a poor woman who under normal circumstances might not have been able to obtain even simple medical care, made people with little experience of clinical research procedures wonder about the motives behind such activities.

Moreover, the research organisation's involvement in HIV research, care and treatment was well-known, and participants involved with the organisation were suspected of being HIV-positive, with the implication of stigma and marital tensions. This was particularly problematic, since the trial consent forms, in conformity with ethical standards, assured participants of confidentiality.

### Alternative identities

This risk of breaching confidentiality or of conflicts with husbands and other family members was highest when individual staff visited participants at home. Most participants had discovered their HIV status upon entry to the study and were struggling with the repercussions. Despite improvements in the perception of HIV in Kenya, disclosure of one's HIV status – reiterated like a mantra in encounters between staff and participants – was difficult for vulnerable young participants, who often hid drugs and study documents from husbands and other relatives (and whose disclosure in other cases led to marital break-up). This was an obstacle for follow-up staff who, when conducting pill counts or examinations, had to be mindful not to unintentionally reveal a participant's HIV status.

Several staff had been chased by angry husbands, or found participants fleeing as they approached; by contrast: ‘for those mothers who had disclosed getting into their homes is not difficult because everyone knows why we are here’. This is why follow-up staff, like Agwata, hid their identity cards, left the vehicle behind or even travelled with unmarked public transport cars, in order to fashion alternative identities or attract less attention. These could be closer to truth – passing as malaria researchers – or less so – claiming to be Church friends or missionaries, or, most commonly, sisters, sisters-in-law or friends. Rather than being a general policy of the research management, these aliases, together with the most appropriate strategies for visiting specific homes, were conceived of by follow-up staff, often together with the participants. These identities had to be maintained over the course of the trial:

‘It was really a challenge because most of them have not disclosed, so if you go to a home at times you are a sister to the participant, at times you are a church group member, or you are trying to prevent malaria…!’.

Some staff expressed that they felt uncomfortable with ‘lying’ about who they were, but that it was the only way to protect the participant and get the work done.

The play with alterative identities was sometimes rather transparent. In Agwata's case, this was taken to the extreme: a well-dressed woman, accompanied by two anthropologist (one of them expatriate), travelling in a big car with driver and red number plates – a sister in law? But even without the presence of an obvious foreigner – which the trial management explicitly avoided during follow-up – fieldwork vehicles carried the red diplomatic number plate and logo of KEMRI/CDC. And although follow-up staff attributed importance to not openly identifying with HIV research, they sometimes chatted among themselves, but in the presence of community members, about their employers, without concern for their incognito. Whatever people implicitly might have wondered about who these strangers really were, explicit conflicts were avoided in this way.

### Decisions about fieldwork strategies

As noted above, responses to the challenges of field encounters were not determined by fixed standard procedures, but relied on spontaneous, ad-hoc decisions by involved staff. If an inebriated husband enters the house of a participant during an adherence survey, common sense guides one to minimise risks for the participant and the staff herself; if one arrives at a participants' house after a long journey only to find it full of visiting in-laws, HIV researcher is not an appropriate social role to perform; and if the participant welcomes one as a stranger, or as a childhood relation, one is advised to play along. Often, decisions had to be made instantaneously, at best based on a quick exchange of glances between visiting staff or participant and staff member. And once one had assumed an identity, it had to be adhered to for future visits.

Rarely the decision to introduce oneself as non-research-staff was recorded, together with the tension or conflict that gave rise to it, in the hand-written ‘Encounters Log’ through which follow-up staff communicated with each other about practical concerns, including participants' fears of disclosing their HIV status, or hostile husbands and relatives. Rather than committing their personal strategies to writ, it appears staff kept these small decisions in their memory.

Weekly staff meetings with the PI and coordinator had a section for follow-up staff, during which critical encounters with husbands and relatives were reported: ‘(study number) had dirty pills during her last visit, she reported that the husband was in denial about her status and had kicked the drugs. She reported that she was not able to bring her husband along’, or ‘(study number) had 4 pills less of both study drugs; she reported that she had a fight with the husband who threw away the drugs.’ Yet, the meetings minutes did not record implications for future encounters, beyond recommending that staff should visit the home in question only in groups, that the situation should be discussed with the participant during a clinical visit, or that the participant's disclosure should be encouraged. The decision to ‘hide’ one's involvement in research was only once discussed in a meeting we attended – on the occasion of a particularly difficult husband and accompanied with a lot of laughter – and it does not feature in the regular meeting minutes, although it was part of most of the home visits we took part in. The necessity to avoid conflicts and protect participants was obvious enough without explication and belonged (together with similarly personal decisions such as giving small gifts to needy participants), to the realm of personal morality and common sense. In the formal procedures of the research, there was no clear space for them, and senior staff or PIs were not commonly involved, except for large decisions, such as recommending use of public transport means instead of recognisable project cars to maintain confidentiality.

### Sisters'

Yet, introducing oneself as close relative or sister was not merely about hiding institutional affiliations and maintaining HIV confidentiality. It also pointed to an important dimension of follow-up work: the forging of close personal relationships during many encounters, often in the privacy of the home, over several years. To the participants, follow-up staff were much more than ‘tools’ of the trial; they talked about ‘feeling nice’ when visited, and praised ‘their’ follow-up staff: ‘my follow-up staff was so good: she washed me when I was bed ridden’. Both staff and the mothers evoked ‘love’ to describe this special bond: ‘my follow-up staff she really loved me’, or: ‘I loved that baby so much, I hope he is doing well now the study is over’. Motherly praise of babies' beauty, fatness or skin, was applied to all trial babies – ‘our babies’ – beyond the professional pride in achieving a negative status for most babies; as one staff said: ‘the positive babies are even the most beautiful and healthy’.

These intimate bonds grew from sharing time and vital events – HIV diagnosis, pregnancy and birth (often the first one), concerns about marital issues, sickness – and from seeing one another in a range of different places – research clinic and public hospital, city house and village home. Follow-up staff (as well as, in many cases, clinicians, nurses and counsellors) became confidants of the women during a period where other relations were strained or ruptured. Participants shared with the female and low-ranking follow-up staff intimate problems that they did not want to tell study clinicians, e.g. concerning violent husbands, disclosure and sexuality. Staff advice was accordingly ‘sisterly’, but no less crucial to the success of the trial: ‘with condoms you need to take it slowly, maybe just leave it under the pillow, or pack in his travelling suitcase’.

These relations, and the opportunities that they afforded to have a positive impact on participants' lives and to save babies from HIV infection, were fulfilling to staff, who reported in interviews high levels of job satisfaction and some of whom, after the end of the study, looked back with nostalgia to the demanding follow-up work: ‘we were active – we would finish work at 7pm and not mind.’

### Strained relations

What staff enjoyed as ‘the humanness’ of follow-up work, the intimacy, flexibility, and responsibility entailed – requiring improvisation and independent decisions, knowledge of specific people and personal judgement – became a burden when participants did not collaborate – ‘she really frustrated me when she missed taking her drugs!’ – or had other things to do:

‘there were some participants who were very friendly. When you go their house they welcome you, they talk to you, they take instructions and put them into practice. But at some point you'll not be friends. You go there, she just continues doing her work, she does not understand that you are also doing your work.’

Follow-up staff's irritation in such situations was professional – concerns with loss to follow-up, treatment efficacy and data validity, and managers' expectations – but also personal, worrying, like any sister, about the well-being of participant and child, and struggling with the perceived ‘stubbornness’, with which participants ignored what to staff seemed obviously beneficial advice.

Similar mixed emotions came into play when staff confronted participants' difficult lives. Getting involved in marital disputes strained sympathies. Blatant poverty pushed the limits of responsibility. More than other staff members, follow-up staff confronted participants' hunger – a particular problem for HIV research. Participants, admonished to take their HIV drugs, countered: ‘when I take these drugs on an empty stomach I feel dizzy and they make me tired so I shall stop taking them.’^11^ Beyond interfering with the needs of the study, hunger also constituted a personal moral challenge: ‘you cannot go without assisting, because you have that human heart to give something small – but we didn't encourage that.’ Small gifts of money or food appeared to be common practice among follow-up staff, and expected by participants, in a cultural context where human relations are premised upon a continuous stream of material transfers and sharing.^12^ But although staff shared the moral values of giving, the gifts were a strain on their resources – ‘you can't help everyone, we also have our limits’ – and they felt helpless in the face of needs they could not satisfy. As ‘sisters’ of sorts, follow-up staff were obliged to help, and often happy enough to be able to make a difference – but then who has 50 plus sisters to care for?

Staff's financial limits aside, the small payments reveal the inevitable discrepancy between ‘humane’ social relations, based on intimacy, specificity and common sense, and the scientific relations between staff and participants in a transnational clinical trial: although these gifts were experienced as a natural part of good human relations, and contributed to positive trial relations and successful data collection, they were not regulated by formal ‘standard operation procedures’ or reported at staff meetings (nor reimbursed from research funds); instead they had to be decided upon ad hoc, by research staff. This gap between formal and informal applies also to the alternative identities described above, or to sisterly advice on how to deal with husbands' mores. Everyone involved in this long-term clinical trial has to deal with this gap – lead scientists who in addition to doing good science want to help needy individuals, or clinicians who in addition to collecting data want to live up to their oath and morality. Follow-up staff bridging day to day the gap between clinic and home, regulated science and the irregularity of life under adverse conditions, bear the brunt of this tension.

## Conclusions

Recent ethnographies of clinical research in Africa have described the importance of ‘relatedness’ to the successful conduct of trials.^13^ They have also revealed the tensions between such relatedness and the scientific and ethical rules of clinical trial work, and at the risks of mixing professional and private, erasing the ‘ordered separations upon which formal ethics and scientific evidence rest’. Moreover, they have pointed at challenges for staff identity, for example when staff who have engaged kinship-like relations over years, disappear after the end of a particular trial, or meet participants off duty,^14^ and they have discussed the wider ethical import of ambiguous researcher identities under conditions of political, economic and educational inequality.

In this paper, we looked at the challenges of identity and relatedness in an HIV research project. We found that follow up staff, because of community perceptions of research, and of concerns with HIV confidentiality, often resorted to alternative identities, presenting themselves as relatives rather than researchers. Such identity switching is necessary to protect participants while producing valid scientific data of great public health importance. It shows that there are situations when complete transparency about one's identity would imply serious ethical breaches. Apart from being a matter of balancing concerns with confidentiality and transparency in research ethics, this matter also raises issues of personal morality and professional practice for the research staff, which must be considered, among other fieldwork challenges, by supervisors and trainers.

Our second main finding was that the assumed identity as a relative was not merely a subterfuge, but described the close relationships between staff and participants under challenging conditions. These close relations, and the responsibilities and abilities to act that they entailed, were valued by staff and participants alike, and contributed to the success of this trial – in terms of patient retention, data validity, and intervention outcomes. This notwithstanding, the expectations and responsibilities – under conditions of stark poverty – entailed by sisterly bonds also proved challenging for the follow-up staff, both emotionally and financially. While the nature of the HIV study we followed produced particularly intimate and long-lasting bonds, similarly close relations – with the attendant tensions – are formed in other types of population-based medical research under similar economic conditions.^15^

These observations concerning identities and relations in HIV research deserve consideration by trial managers and ethicists. To negotiate practical – as well as ethical – concerns with confidentiality and stigma on the one hand; and the social and moral commitment of human relations on the other, requires openness and flexibility: possibilities for staff to develop relationships in meaningful and responsible ways; and opportunities within medical research settings to articulate the complex and equivocal challenges arising from personal encounters across the dividing line between researchers and researched.

These observations within HIV research follow-up staff have relevance beyond clinical trials. The expansion of HIV care and treatment, and community-based and home-based HIV interventions, supported by large-scale donors and often involving scientific and academic organisations like KEMRI and CDC, is shaped by the particular importance of strict treatment adherence in HIV care, as well as by the requirements of continual evaluation, which requires close patient supervision and surveillance. In this context, community follow-up has become a salient feature of interventions, which helps to ascertain treatment adherence and intervention success, as well as to justify funding and support technical innovation. Although community follow up has been used earlier, e.g. in the context of leprosy or TB interventions, it has expanded to an unprecedented scale by today's HIV interventions in Africa (and attendant interventions, e.g. against TB, funded by similar donors and shaped by similar premises). While patients in Kenya still have to seek help, often in vain, for most other medical problems (including many health problems indirectly associated to HIV), patients in large-scale HIV interventions experience, like the clinical trial participants above, the inverted directionality of health care described above: follow-up staff – defaulter tracers, clinic and community health assistants, community liaison, peer mobilisers etc. – follow them actively, in order to ensure adherence and clinic visits. The observations about identity and relationality, above, might thus have relevance for this large new cadre of health care staff.

## Footnotes

1 See e.g. E. Kalipeni, S. Craddock, et al. 2004. *HIV and AIDS in Africa: beyond epidemiology*. Oxford: Blackwell

2 See B.G. Schoepf. Ethical, methodological and political issues of AIDS research in Central Africa. *SocSciMed* 1991; 33(7): 749–763; B.G. Schoepf. “International AIDS Research in Anthropology: Taking a Critical Perspective on the Crisis. *Annual Review of Anthropology* 2001; 30: 335–361; M.D. April. Rethinking HIV exceptionalism: the ethics of opt-out HIV testing in sub-Saharan Africa. *Bulletin of the World Health Organisation* 2010; 88: 703–708.

3 J. Stadler & E. Saethre. Rumours about blood and reimbursements in a microbicide gel trial. *African Journal of AIDS Research* 2011; 9(4): 345–353; see also S.R. Whyte, M.A. Whyte & D. Kyaddondo. Health workers entangled: confidentiality and certification. In: *Morality, Hope and Grief. Anthropologies of AIDS in Africa.* H.J. Dilger & U. Luig (eds). Oxford: Berghahn, pp. 80.101.

4 C. Simon & M. Mosavel. Getting personal: Ethics and identity in global health research. *Dev World Bioeth* 2011; 11(2): 82–92.

5 C.S. Molyneux, W. Mutemi, et al. Trust and informed consent: insights from community members on the Kenyan Coast. *SocSciMed* 2005; 61: 1463–1473; J. Fairhead, M. Leach, et al. Where techno-science meets poverty: medical research and the economy of blood in The Gambia, West Africa. *Soc Sci Med* 2006; 63(4): 1109–1120; P.W. Geissler, A. Kelly, et al. ‘He is now like a brother, I can even give him some blood’ – relational ethics and material exchanges in a malaria vaccine ‘trial community’ in The Gambia. *SocSciMed* 2008; 67(5): 696– 707; C. Gikonyo, P. Bejon, et al. Taking social relationships seriously: Lessons learned from the informed consent practices of a vaccine trial on the Kenyan Coast. *SocSciMed* 2008; 67(5): 708–720. A.H. Kelly, M. Pinder, D. Ameh, S. Majambere, & S. Lindsay. ‘Like Sugar and Honey': The embedded ethics of a larval control project in The Gambia, *SocSciMed* 2010; 70(12): 1912–1919.

6 T.K. Thomas, R. Masaba, et al. Triple-Antiretroviral Prophylaxis to Prevent Mother-To-Child HIV Transmission through Breastfeeding – The Kisumu Breastfeeding Study, Kenya: A Clinical Trial. *PLoS Med* 2011; 8(3): e1001015.

7 Through training opportunities provided by the research organisation all follow-up staff obtained diploma level in subjects such as community health and counselling during the course of the study.

8 Names and circumstances have been changed for anonymity. Agwata's story is a composite of several stories.

9 P.W. Geissler. *Kachinja* are coming: Encounters around a medical research project in a Kenyan village. *Africa* 2005; 75(2): 173–202; P.W. Geissler & R. Pool. Popular concerns with medical research projects in Africa – a critical voice in debates about overseas research ethics. *Trop Med Int Health* 2006; 11(7): 975–982; Fairhead & Leach op.cit. note 5; Stadler & Saethre *op. cit.* note 3.

10 Geissler, ibid.

11 R.J. Prince. HIV and the Moral Economy of Survival in an East African City. *Medical Anthropology Quarterly* 2012; 26(4): 534–556.

12 Ibid. See P.W. Geissler & R.J. Prince. 2010. *The Land is Dying. Contingency, Creativity and Conflict in Western Kenya*. Oxford, New York: Berghahn; P. Shipton, P. Debts and trespasses: land, mortgages and the ancestors in western Kenya. *Africa* 1992; 62(3): 357–388.

13 See e.g. Molyneux et al. op.cit.; Fairhead et al. op.cit.; Geissler et al. op.cit; Gikonyo et al. op.cit.); P.W. Geissler. 2011. Studying Trial Communities: Anthropological and Historical Inquiries into Ethos, Politics and Economy of Medical Research in Africa. In: *Evidence, Ethos and Ethnography: The Anthropology and History of Medical Research in Africa*. P. W. Geissler and C. Molyneux (eds). Oxford: Berghahn: 1–28.

14 A. Kelly, 2011. Remember Bambali: Evidence, Ethics and the Co-production of Truth. In: *Evidence, Ethos and Ethnography: The Anthropology and History of Medical Research in Africa*. P. W. Geissler and C. Molyneux (eds). Oxford: Berghahn: 229–244.

15 See e.g. Geissler et al. 2008, op.cit.

## Biographies

**Philister Adhiambo Madiega** works for the Kenyan Medical Research Institute (KEMRI) in Kisumu, Kenya, in the KEMRI collaboration with the Centres for Disease Control and Prevention (CDC). Previously, she worked for several years in ethnographic field studies and wrote her MSc dissertation on HIV care and treatment support staff.

**Gemma Jones** is currently finishing her doctorate in social anthropology at the London School of Hygiene, Anthropologies of African Biosciences Research Group. Her work explores how medical research figures in the day-to-day lives of those involved as researchers and participants in transnational medical research in rural Western Kenya.

**Ruth Jane Prince** is Mellon Fellow in Science, Medicine and Society in Africa at the University of Cambridge. Together with Wenzel Geissler she published the monograph *The Land is Dying* (2010). Her edited book (with Rebecca Marsland), ‘*Making Public Health in Africa: Ethnographic and Historical Perspectives*’ will be published in 2013 with Ohio University Press.

**Paul Wenzel Geissler** teaches social anthropology at the Institute of Social Anthropology, University of Oslo, and the London School of Hygiene. His research explores the practice of scientific research and collaboration in Africa, combining ethnography and history. His most recent book is *Evidence, Ethos and Experiment: the anthropology and history of medical research in Africa* (2011; edited with Sassy Molyneux).

